# Nurses’ Self-Efficacy, Confidence and Interaction With Patients With COVID-19: A Cross-Sectional Study

**DOI:** 10.1017/dmp.2021.1

**Published:** 2021-01-07

**Authors:** Loai Abu Sharour, Ayman Bani Salameh, Khaled Suleiman, Maha Subih, Mamdouh EL-hneiti, Mahmoud AL-Husaami, Khloud Al Dameery, Omor Al Omari

**Affiliations:** 1 Faculty of Nursing, AL-Zaytoonah University of Jordan (ZUJ), Amman, Jordan; 2 School of Nursing, University of Jordan, Amman, Jordan; 3 Faculty of Nursing, Sultan Qaboos University, Al-khod, Sultanate of Oman

**Keywords:** self-efficacy, self-confidence, nurse-patient interaction, COVID-19, nurses

## Abstract

**Objective:**

The aim was to evaluate nurses’ self-efficacy, confidence, and nurse-patient interaction during caring of patients with coronavirus disease 2019 (COVID-19).

**Methods:**

A cross-sectional design with online survey was used with a Self-efficacy scale, Self-confidence scale, and Caring nurse-patient interaction scale: 23-item Version-Nurse (CNPI-23 N).

**Results:**

A sample of 120 nurses participated in the current study. The results showed that the participants had a moderate level of self-efficacy, self-confidence and interaction (M = 28.84 (SD = 7.7), M = 47.41 (SD = 9.0), and M = 93.59 (SD = 16.3), respectively). Positive relationships were found between nurse’ self-efficacy, self-confidence, and nurse-patient interaction (r = 0.81; *P* < 0.0001 and 0.79; *P* < 0.0001, respectively). Significant differences were found in self-efficacy according to years of experience, academic qualifications and position (F = 2.10; *P* = 0.003; F = 3.60; *P* = 0.002, and F = 2.60; *P* =0.007, respectively). Furthermore, the results indicated that there was a significant difference in self-confidence and nurse-patient interaction also.

**Conclusion:**

Nurse educators and administrators should develop and implement further strategies, such as continuing education and training, compensatory payment, organizational support, and availability of protective measures to increase their self-efficacy, self-confidence, and interaction with COVID-19 patients.

In December 2019, a highly infectious virus was identified by the scientists and experts in Wuhan, the capital of China’s Hubei province. This new coronavirus (severe acute respiratory syndrome coronavirus 2 [SARS-CoV-2], the cause of the disease called COVID-19) became an unexpected world health crisis and was classified by the World Health Organization (WHO) as a pandemic.^1^ Currently (December 2020), more than 77 million patients have been diagnosed with COVID-19 worldwide and almost 1.7 million deaths have occurred, and numbers are expected to increase.

Patients with COVID-19 experience flu like symptoms, including sneezing, coughing, running nose, fever, general weakness, and difficulty in breathing.^2^ The exponentially growing number of cases and deaths increase pressure on the health-care system in affected countries. Health-care workers (HCW) are also experiencing huge pressures.^3^ At this moment, health-care professionals, including doctors, nurses, scientists, paramedics, laboratory staff, radiologists, physiotherapists, and microbiologists, around the world are struggling to manage the alarming increasing number of cases. Lack of knowledge about COVID-19 transmission, fear of outbreak, pressure from fearful family members, lack of adequate and available personal protective measures and equipment, and no clear curable treatment plan are major problems. Furthermore, HCWs are at high risk of infection because of the nature of their job, as they are physically very close to patients and in direct contact with them during medical assessment and intervention.^4^ This might affect their self-efficacy, confidence, and interaction with COVID-19 patients.

Nurse-patient interaction is a prominent factor playing an influential role in the patient experience of care provided by bedside nurses.^5,6^ It consists of the appropriate attitude and behaviors that cover the clinical, relational, and humanistic domains of nursing. The nurse-patient interaction was linked positively to the quality of nursing care,^7,8^ increasing the feeling of being cared for,^9^ and increasing patient involvement in the treatment plan, power, satisfaction, coping^10-12^ and self-transcendence.^13^

Self-efficacy is a well-known concept, it affects the nurses’ beliefs, actions and behaviors while caring for sick patients.^14^ Experts and scientists recognize self-efficacy as a powerful variable that affects the nurses’ motivation to care, thinking processes and decision making, prioritizing interventions, and encouraging them to continue caring for the patients despite difficulties and failure.^15-18^

Self-confidence is a powerful factor that influences rapid, appropriate, safe, and accurate nursing intervention in an emergency situation and also while caring for critically ill patients.^19^ Nurses with higher levels of self-confidence show more competence in developing appropriate and safe interventions, making correct decisions, and providing patients with a better quality of care.^20^

Caring for patients during a pandemic period, such as COVID-19, is a challenge for all health professionals, especially the nurses. To increase nurses’ abilities to provide optimal care to COVID-19 patients, it is important to evaluate their level of self-efficacy, confidence, and interaction. The results can be used by health-care administrators to develop strategies or modules to maximize these influential variables. This study was conducted to evaluate self-efficacy, self-confidence, and nurse-patient interaction during caring of patient with COVID-19 in Jordan.

## Methods

A cross-sectional and correlational design was used with an on-line survey in the current study. After ethical approval was granted, the research team structured the on-line survey and double reviewed it before generating its link. Then, the link was uploaded on Facebook and distributed by the research team. Detailed information about study purpose, methods, and instructions to submit the questionnaire were provided at the beginning of the questionnaire. In addition, participants were informed that they were free to participate and withdraw anytime, no identification details were required, and that data would only be used for scientific purpose. Participants who met the inclusion criteria, that is being a Jordanian nurse, having access to the Internet, and working in hospitals, were invited to participate.

The Cohen (1998) formula was used in the current study to estimate the sample size. Cohen identified 3 levels of effect of sample size: small effect 0.20, medium effect 0.50, and large effect 0.80. Based on this classification, a medium effect of correlations between the study variables was used to guide the sample size calculation.^21^ A sample of 115 nurses was estimated with an effect size of 0.5, alpha at 0.05, and a power of 0.80. However, a convenience sample of 120 nurses completed the study survey. Link to study survey was republished daily by the research team. Data were collected in a 3-wk period. After the data collection phase was completed, 2 researchers from the team printed out the questionnaires and entered the data into Statistical Package for the Social Sciences (SPSS) (version 26); a third researcher re-checked the data entry for increased accuracy.

### Measures

In addition to sociodemographic characteristics, including gender, age, position, marital status, academic qualifications, and years of experience, 3 instruments were used in the current study including:

*Self-Confidence Scale (SCS):* Contains 12 items with a 5-point Likert scale (1 = not confident; 2 = hardly confident; 3 = confident; 4 = very confident; and 5 = extremely confident), with the scores ranging between 12 and 60, and higher scores indicating more confidence. The scale was developed by Hicks in 2006; it was valid and reliable with Cronbach’s alpha 0.96.^22^

*Self-Efficacy Scale:* It has 10 items with a 4-point Likert scale (1 = not at all true; 2 = hardly true; 3 = moderate true; and 4 = exactly true). The scores ranged between 10 and 40, higher scores indicating a high level of self-efficacy. The scale was developed by Schwarzer and Jerusalem and was a valid and reliable scale with a Cronbach’s alpha of 0.80.^23^

*Caring Nurse-Patient Interaction Scale: 23-Item Version-Nurse (CNPI-23 N):* The CNPI-23N is a short scale of 23 items with a 5-point Likert scale (1 = not at all to 5 = extremely). It reflects 4 main domains of nursing care, including humanistic care (4 items), relational care (7 items), clinical care (9 items), and comforting care (3 items). The scores range between 23 and 115, with higher scores indicating more interaction. The scale was valid and reliable with Alpha coefficients for the 4 domains also adequate (0.63 to 0.74, 0.90 to 0.92, 0.80 to 0.94, and 0.61 to 0.76, respectively).^24^

### Analysis

Descriptive analysis including mean, standard deviation (SD), frequency, and percentage were performed. Pearson moment correlation (r) was used to explore the relationships between the study variables, including self-efficacy, self-confidence, and nurse-patient interaction. Differences in self-efficacy, self-confidence, and nurse-patient interaction according to the nurse’ academic qualifications, years of experience, gender, and positions were evaluated by using an analysis of variance (ANOVA) test. Data were analyzed by using SPSS (version 26).

## Results

### Sample Characteristics

A sample of 120 participants completed the study survey, around half of them were females (*N* = 64, 53.3%), a majority had a bachelor degree (*N* = 87, 72.5%) and were working as registered nurses (*N* = 105, 87.5%). Table 1 details these results.

**Table 1.** Sample characteristics (*N* = 120)

	Variable	Frequency (%)
1	Age (y)	
	22-26	37 (30.8)
	27-30	16 (13.4)
	30-35	37 (30.8)
	36-40	14 (11.6)
	More than 40	16 (13.4)
2	Years in nursing	
	1-5	43 (35.8)
	6-10	33 (27.5)
	More than 10	44 (36.7)
4	Gender	
	Female	64 (53.3)
	Male	56 (46.7)
5	Education level	
	Diploma	10 (8.3)
	Bachelor	87 (72.5)
	Postgraduate	23 (19.2)
6	Current position	
	Registered nurse	105 (87.5)
	Head nurse	15 (12.5)
7	Marital status	
	Single	56 (46.7)
	Married	61 (50.8)
	Divorced	3 (2.5)

### Nurses’ Self-Efficacy, Self-Confidence, and Nurse-Patient Interaction Domains

Mean and SD were calculated. The results showed that the participants had moderate self-efficacy (mean = 28.84, SD = 7.7) and self-confidence (mean = 47.41, SD = 9.0). High Humanistic Care and Comforting Care were reported (mean = 15.93, SD = 2.9; and mean = 12.93, SD = 2.4, respectively). The results are presented in Table 2.

**Table 2.** Mean and SD of study’s variables (*N* = 120)

	Item	Possible range	Minimum	Maximum	Mean (SD)
1	Self-efficacy	10 - 40	20.0	40.0	28.84 (7.7)
2	Self-confidence	12 - 60	24.0	60.0	47.41 (9.0)
3	Total CNPI-23N	23 - 115	55.0	115.0	93.59 (16.3)
4	Clinical care	9 - 45	19.0	45.0	37.0 (7.1)
5	Relational care	7 - 35	13.0	35.0	27.72 (5.3)
6	Humanistic care	4 - 20	8.0	20.0	15.93(2.9)
7	Comforting care	3 - 15	6.0	15.0	12.93 (2.4)

### Relationship Among Self-Efficacy, Self-Confidence, and Nurse-Patient Interaction

Pearson moment correlation (r) was used to explore the relationships among the study variables, including self-efficacy, self-confidence, and nurse-patient interaction. Significant positive relationships were found among self-efficacy, self-confidence, and total CNPI-23 N (r = 0.81, *P* < 0.0001; and r = 0.79, *P* < 0.0001, respectively). Table 3 details these results.

**Table 3.** Relationship between self-efficacy, self-confident, and nurse-patient interaction (*N* = 120)

Variable	Self-efficacy	Self-confidence	Total CNPI-23N	Clinical care	Relational care	Humanistic care	Comforting care
Self-efficacy	1	0.73**	0.81**	0.78**	0.75**	0.71**	0.61**
Self-confidence		1	0.79**	0.76**	0.71**	0.67**	0.63**
Total CNPI-23N			1	0.94**	0.91**	0.89**	0.83**
Clinical care				1	0.76**	0.77**	0.74**
Relational care					1	0.81**	0.70**
Humanistic care						1	0.71**
Comforting care							1

** Correlation is significant at the 0.01 level (2-tailed).

### Differences in Self-Efficacy, Self-Confidence, and Nurse-Patient Interaction According to the Participants’ Characteristics

Differences in the participants’ levels of self-efficacy, self-confidence, and nurse-patient interaction according to their years of experience, gender, academic qualifications, and current positions were calculated by using an ANOVA test. The results indicated that there was a significant difference in self-efficacy according to their years of experience, academic qualifications, and position (F = 2.10, *P* = 0.003; F = 3.60, *P* = 0.002; and F = 2.60, *P* = 0.007, respectively). Furthermore, the results indicated that there was a significant difference in self-confidence and nurse-patient interaction also. Table 4 details these results.

**Table 4.** Self-efficacy, self-confidence, and nurse-patient interaction by demographic characteristics of participants (*N* =120)

Variable	Frequency (%)	Self-efficacy score/40 (SD) *F* (*P*-value)	Self-confidence score/60 (SD) *F* (*P*-value)	Total CNPI-23N score/115 (SD) *F* (*P*-value)
Years in nursing				
1-5	43 (35.8)	22.10 (3.4)	42.10 (2.8)	86.10 (1.2)
6-10	33 (27.5)	24.30 (2.1)	45.10 (1.0)	90.10 (1.7)
More than 10	44 (36.7)	27.10 (1.1)	46.85 (0.8)	92.80 (1.1)
		2.10 (0.003)	3.05 (0.004)	4.80 (0.002)
Gender				
Female	64 (53.3)	27.60 (1.3)	46.10 (1.4)	90.10 (0.7)
Male	56 (46.7)	25.10 (0.71)	45.70 (1.8)	89.20 (1.6)
		3.10 (0.610)	4.05 (0.717)	5.10 (0.810)
Education level				
Diploma	10 (8.3)	23.10 (1.7)	39.05 (2.1)	85.10 (2.1)
Bachelor	87 (72.5)	26.10 (1.8)	45.90 (1.2)	91.10 (1.4)
Postgraduate	23 (19.2)	26.80 (1.1)	46.80 (0.7)	92.75 (0.9)
		3.60 (0.002)	3.80 (0.004)	5.50 (0.003)
Current position				
Registered nurse	105 (87.5)	26.10 (1.8)	45.90 (1.2)	91.10 (1.4)
Head nurse	15 (12.5)	26.80 (1.1)	46.80 (0.7)	92.75 (0.9)
		2.60 (0.007)	3.02 (0.006)	4.20 (0.002)

## Discussion

The current study aimed to evaluate nurses’ self-efficacy, confidence, and nurse-patient interaction while caring for patients with COVID-19 in Jordan. In this study, Jordanian nurses showed good levels of self-efficacy, self-confidence, and nurse-patient interaction while treating these patients. COVID-19 nurse-patient interaction consists of 4 domains of nursing practice, including clinical care, relational care, humanistic care, and comforting care. These domains were maintained and provided by the Jordanian nurses in the current study. The results indicate that the nurses were willing to provide the appropriate care for the patients with COVID-19 and recognized the importance of nurse-patient interaction in this critical condition. A moderate level of nurse-patient interaction was reported in the current study. This moderate level could be explained by fear of contamination, family influence to avoid direct contact for a long time, and workplace environment such as number of assigned patients, available safety measures, and perceived level of knowledge about the pandemic.^25^ These findings contradict 3 previous studies. First, in 2003, during the outbreak of severe acute respiratory syndrome (SARS), many HCWs, such as doctors and nurses, were not willing to treat and interact with SARS patients.^26-28^ Second. in Germany, HCWs, including doctors, nurses, medical students, and administrators, showed their intention to leave their job during the pandemic to protect themselves and their families.^29^ Third, 50% of clinical and nonclinical HCWs were unwilling to work during the pandemic in the United States.^30^

In Jordan, many strategies were implemented to enhance HCWs especially nurses’ self-efficacy, self-confidence, and nurse-patient interaction during COVID-19 pandemic, including increasing their knowledge about the disease, continuing education and training, reinforcement of the positive attitudes, compensation payment, verbal and nonverbal expressions of thanks by the government and the public, availability of personal protective equipment (PPE), decreased workload and nurse-patient ratio, and continuing psychological support.

In Jordan, self-efficacy and self-confidence played a significant and influential role in increasing nurse-patient interaction during the COVID-19 pandemic. Self-efficacy was linked to the nurses’ positive attitude and health behaviors, such as interaction with the patients in different and difficult circumstance and conditions.^31^ During the previous pandemic of SARS, nurse self-efficacy was identified as a main predictor and influential factor for nurses’ intention to care for and interact with patients with SARS in Taiwan.^32^ Self-confidence has been associated with mastery of clinical skills and increased clinical proficiency and competency.^33^ In the current study, Jordanian nurses showed self-confidence in treating and interacting with patients with COVID-19; this could be explained and linked to the preparation and training, availability of the protective measures, and organizational support. During the pandemic, a confident nurse could master his/her clinical skills, communicate and interact with the patients, and be able to respond to emerging situations properly and effectively.^34^

As frontline HCWs, workload during the pandemic is considered a challenge for the nurses and requires special and additional skills and knowledge. Results from the current study indicate that nurses with more years of experience, who had postgraduate degrees, and who worked as head nurses showed high levels of self-efficacy, self-confidence, and better interaction while caring for patients with COVID-19. These results are not surprising as the senior and highly educated nurses are more competent in using evidence-based practice, taking responsibility, controlling and coping with complex situations, working within the team effectively, having leadership roles, and maintaining the balance between the patients’ demands and HCWs’ health.^35^ During the influenza pandemic, American and Australian senior nurses showed more competence, confidence, and rapid response to the complex situations, coordination of care, and collaboration with other HCWs.^35,36^

Two limitations in the current study include, first, a self-reporting questionnaire was used to evaluate the nurse-patient interaction. This might not be the best way to evaluate the actual interaction; future research can be conducted using different research methods, such as observation. Second, an online-survey was used in the current study due to quarantine conditions; this requires access to the Internet. Therefore, nurses with limited access to the Internet were unable to participate. Further research using a paper-based questionnaire is recommended.

## Conclusions

Working during the pandemic and outbreak of COVID-19 is challenging and stressful for nurses. Increasing their self-efficacy and self-confidence will increase their interaction with and treatment of patients with COVID-19. Nurse educators and administrators should develop and implement further strategies, such as continuing education and training, compensation payment, organizational support, and availability of protective measures to improve their self-efficacy, self-confidence, and interaction with COVID-19 patients.
