# Nurses’ Self-Efficacy, Confidence and Interaction with Patients with COVID-19: A Cross-Sectional Study – Corrigendum

**DOI:** 10.1017/dmp.2021.96

**Published:** 2021-08-18

**Authors:** Loai Abu Sharour, Ayman Bani Salameh, Khaled Suleiman, Maha Subih, Mamdouh EL-hneiti, Mahmoud AL-Hussami, Khloud Al Dameery, Omor Al Omari

**Keywords:** self-efficacy, self-confidence, nurse-patient interaction, COVID-19, nurses, corrigendum

There was an error in the spelling of author Mahmoud AL-Hussami’s name in the original publication of this article.^1^ The article has since been corrected.
